# Tobacco smoking and trends in histological subtypes of female lung cancer at the Cancer Hospital of the Chinese Academy of Medical Sciences over 13 years

**DOI:** 10.1111/1759-7714.13141

**Published:** 2019-07-10

**Authors:** Qiang Zeng, Emily Vogtmann, Man‐man Jia, Mark Parascandola, Ji‐bin Li, Yan‐ling Wu, Qin‐fu Feng, Xiao‐nong Zou

**Affiliations:** ^1^ Department of Radiation Oncology, National Cancer Center/National Clinical Research Center for Cancer/Cancer Hospital Chinese Academy of Medical Sciences and Peking Union Medical College Beijing China; ^2^ Metabolic Epidemiology Branch National Cancer Institute Division of Cancer Epidemiology and Genetics, Bethesda Maryland USA; ^3^ Department of Gynecological Oncology Henan Cancer Hospital Zhengzhou China; ^4^ Tobacco Control Research Branch National Cancer Institute Division of Cancer Control and Population Sciences, Bethesda, Maryland USA; ^5^ Office of Cancer Registry, National Cancer Center/National Clinical Research Center for Cancer/Cancer Hospital Chinese Academy of Medical Sciences and Peking Union Medical College Beijing China

**Keywords:** female, histology, Lung neoplasms, smoking, trends

## Abstract

**Background:**

Lung cancer is the leading cause of cancer mortality among women in China, and incidence and mortality continue to rise despite the fact that smoking prevalence is very low among Chinese women.

**Aim:**

This study investigated tobacco smoking and trends in histological subtypes of female lung cancer in a central cancer hospital in China.

**Methods:**

Demographic, smoking history and histological information on female lung cancer patients diagnosed or treated from 2000 to 2012 was collected from the Cancer Hospital, Chinese Academy of Medical Science (CHCAMS). The classification of histological subtypes and clinical stages were conducted using the ICD‐O‐3 and Eighth AJCC Cancer Staging Manuals. Time‐trends of histological subtypes were analyzed based on annual percentage change (APC).

**Results:**

Overall, 5870 female cases of lung cancer were included in the analysis. The number of female lung cancer patients increased from 509 (2000–2002) to 1744 (2011–2012). The most common histological type of lung cancer was adenocarcinoma (ADC) (72.93%), followed by small cell lung cancer (SCLC) (11.06%), squamous cell carcinoma (SCC) (8.38%) and other (7.63%). Among smokers, the proportion of SCC decreased from 40.5% to 23.7% (*P* = 0.005), while ADC increased from 35.7% to 50.7% (P = 0.009). In non‐smokers, ADC increased from 63.1% to 80.6% (P = 0.006) and SCC decreased from 13.6% to 4.5% (*P* = 0.016). Among SCC cases, smokers made up a larger proportion of early stage (I/II: 47.1%) compared with late stages (III, 34.3%; IV, 18.6%).

**Conclusion:**

The number of female lung cancer patients has increased in CHCAMS. In both smoking and non‐smoking cases, the proportion of adenocarcinoma increased. Squamous cell carcinomas were more likely to be diagnosed in early stages among smokers.

## Introduction

Lung cancer is the leading cause of cancer death among both men and women in China, and its incidence among women is second only to breast cancer.^1^, ^2^ In 2015, there were 267 000 new lung cancer diagnoses (15% of all new female cancers) and 197 000 lung cancer deaths (23% of all female cancer deaths) among women in China.^1^, ^3^, ^4^ Lung cancer incidence and mortality has been increasing among both Chinese men and women.^5^, ^6^ While lung cancer is the most strongly associated of all smoking‐related diseases^7^, ^8^, ^9^, ^10^ and smoking is estimated to account for over 80% of lung cancers worldwide,^11^ smoking prevalence among Chinese women remains very low. In 2015, smoking prevalence in China was 52.1% for men and 2.7% for women.^12^ However, China has over 315 million cigarette smokers and accounts for over 40% of the world’s tobacco consumption, and many non‐smoking women are exposed to secondhand tobacco smoke.^13^

Our previous studies on both male and female patients at the Cancer Hospital, Chinese Academy of Medical Science (CHCAMS) found differences in lung cancer patterns and histologic subtypes over the past decade.^14^, ^15^, ^16^Among men, the vast majority (83%) of lung cancers occurred in smokers. Among male lung cancer patients who smoked, the main lung cancer histological type was squamous cell carcinoma (SCC) (39.38%), followed by adenocarcinoma (ADC) (29.85%). In contrast, among non‐smoking patients, ADC was 53.86% and SCC was 16.64%. ADC increased from 38.03% to 67.83%. Among female lung cancer patients, the major histological subtype was ADC (65.79%), followed by SCC (10.21%). However, all groups (men and women, smokers and non‐smokers) saw substantial increases in ADC and decreases in SCC between 2000 and 2012.

Given the different characteristics in lung cancer histology by gender and smoking status, it is important to understand the impact of smoking status on lung cancer histology in women in China. Previous studies showed the effect of smoking on lung cancer histology in male lung cancer cases but a similar analysis has not yet been completed for female lung cancer patients. In the present study, we investigated tobacco exposure in relation to female lung cancer in China, using data from the CHCAMS.

## Methods

### Study subjects

We included data from female patients with lung cancer (ICD10: C34),^17^ diagnosed or treated in the Chinese Academy of Medical Sciences in the period from 1 January 2000 to December 31 2012. The cases were classified into histological subtypes using data from medical records, SCC，ADC，SCLC, large cell lung cancer (LCLC), adenosquamous carcinoma (ASC), other specified lung cancer, or non‐specified. Data regarding demographics (i.e., gender, date of birth, and place of residence) and smoking history (i.e., current smoking status and age at smoking initiation) were also extracted from the medical records.

### Definition and classification

#### Smoking status

Ever smokers were defined as those who had smoked at least 100 cigarettes (or equivalent amount of tobacco) in their lifetime. Never‐smokers were those who either had never smoked at all or had never been daily smokers and smoked less than 100 cigarettes (or equivalent amount of tobacco) in their lifetime.

#### Lifetime smoking exposure

Total pack‐years was used to estimate lifetime smoking exposure by averaging the number of daily packs of cigarettes smoked multiplied by total years of smoking.

#### Clinic stage

The categories of clinical stage were assigned to each case using the Eighth AJCC Cancer Staging Manual from the American Joint Committee on Cancer (AJCC).^18^

### Statistical analysis

SAS 9.3 was used to calculate the distribution and test the statistical differences in ages, histological subtypes, and residential areas between the smoking and non‐smoking patients. Relative frequencies (RF) of the most frequent histological subtypes/groups of lung cancer were calculated by year of diagnosis, residential area, clinical stage and amount of smoking. The annual percentage change was used to estimate the time period differences using the joinpoint software model (4.3.1.0.). Chi‐ square test and *Z*‐test were used for differences tests between smoking and non‐smoking patients in major histologic subtypes, and *P* < 0.05 was considered as statistically significant.

## Results

There were 7142 female lung cancer cases identified from the health information system. Data from 2000 to 2012 were collected and patient identification numbers used in order to achieve anonymity. The cases with valid and complete information on histology and demography were selected after duplicates were deleted, as well asmissing values in histology, date of birth, date of diagnosis, and smoking history. Finally, a total of 5870 cases of Chinese female lung cancer were selected as study subjects. Over one‐third of patients came from Beijing (38.1% of smokers and 34.9% of non‐smokers) while the rest came from surrounding provinces. 5240 (89.3%) were non‐smokers and 630 were ever smokers (10.7%), as demonstrated in Table 1. ADC was the most common histologic subtype, accounting for more than two‐thirds of cases overall.

**Table 1:** General characteristics of study patients

	All		Smokers		Non‐smokers			
	N	%	N	%	N	%	*X* ^2^	*P*
Total	5870	100	630	10.7	5240	89.3		
Age (years)							281.84	< 0.001
< 40	354	6.0	11	1.8	343	6.6		
40–49	1030	17.5	43	6.8	987	18.8		
50–59	1933	32.9	114	18.1	1819	34.7		
60–69	1812	30.9	287	45.6	1525	29.1		
≥ 70	741	12.6	175	27.8	566	10.8		
Year of diagnosis							47.76	< 0.001
2000–2002	509	8.7	84	13.3	425	8.1		
2003–2004	415	7.1	39	6.2	376	7.2		
2005–2006	786	13.4	79	12.5	707	13.5		
2007–2008	1003	17.1	130	20.6	873	16.7		
2009–2010	1413	24.1	146	23.2	1267	24.2		
2011–2012	1744	29.7	152	24.1	1592	30.4		
Histologic subtype							464.87	< 0.001
SCC	492	8.4	183	29.1	309	5.9		
ADC	4281	72.9	295	46.8	3986	76.1		
SCLC	649	11.1	114	18.1	535	10.2		
LCC	177	3.0	22	3.5	155	3		
ASC	137	2.3	10	1.6	127	2.4		
Other	134	2.3	6	1	128	2.4		
Residential area							92.81	< 0.001
Beijing	2068	35.2	240	38.1	1828	34.9		
Hebei	839	14.3	85	13.5	754	14.4		
Inner Mongolia	495	8.4	71	11.3	424	8.1		
Heilongjiang	443	7.5	89	14.1	354	6.8		
Liaoning	403	6.9	51	8.1	352	6.7		
Shandong	368	6.3	26	4.1	342	6.5		
Other	1254	21.4	68	10.8	1186	22.6		

ADC, adenocarcinoma; ASC, adenosquamous carcinoma; LCC, large cell carcinoma; SCC, squamous cell carcinoma; SCLC, small cell lung cancer.

Among smokers, the majority of lung cancer diagnoses occurred among women aged 60–69 years (287, 45.6%) or 75 years and older (175, 27.8%). The common histologic subtypes were ADC (295, 46.8%), SCC (183, 29.1%), and SCLC (114, 18.1%). Among non‐smokers, the majority of lung cancer diagnoses occurred among women aged 50–59 years (1819, 34.7%) or 60–69 years (1525, 29.7%), and the most common histologic subtypes were ADC (3986, 76.1%) and SCLC (535, 10.2%).

### Trends in histologic subtypes

**Figure 1:**
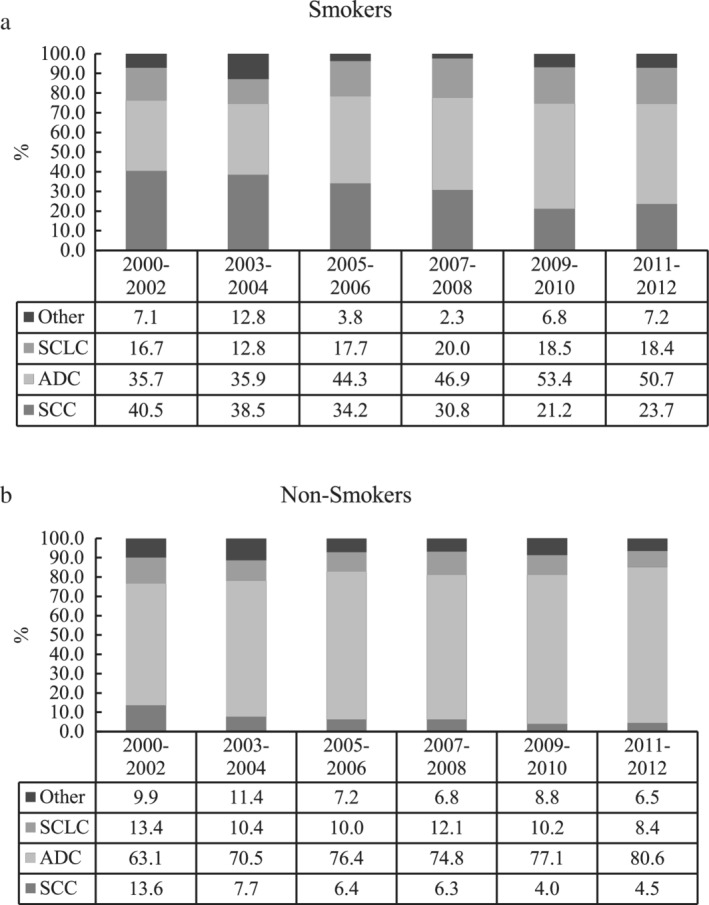
Changes of histology in smoking and non‐smoking patients from 2000 to 2012. APC in smoking lung cancer patients from 2000 to 2012. (**a**) Smokers: SCC, APC = −11.7%, *P* = 0.005; ADC, APC = 8.6%, *P* = 0.009; SCLC, APC = 4.4%, *P* = 0.234; Other, APC = −8.2%; *P* = 0.517; (**b**) Nonsmokers: SCC, APC = −21.33%, *P* = 0.006; ADC, APC = 3.9%, *P* = 0.016; SCLC, APC = −6.1%, *P* = 0.107; Other: APC = −8.23%, *P* = 0.122. 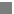 SCC, 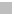 ADC, 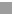
SCLC, 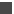 Other

The distribution of histological subtypes changed over time among both smokers and non‐smokers. Figure 1 shows the relative frequencies (RF) of major histologic subtypes among smoking patients (i) and non‐smoking patients (ii) from 2000 to 2012. In the smoking group, the RF of SCC decreased from 40.5% in 2000–2002 to 23.7% in 2011–2012 (APC = −11.68%, *P* = 0.005), while the RF of ADC increased from 35.7% to 50.7% (APC = 8.63%, *P* = 0.009) in the same time period. In the non‐smoking group, the RF of ADC increased from 63.1% in 2000–2002 to 80.6% in 2011–2012 (APC = 3.86%, *P* = 0.016), while the RF of SCC decreased from 13.6% to 4.5% (APC = −21.33%, *P* = 0.006). Thus, while the proportion of ADC to SCC was greater among non‐smoking compared with smoking cases, the trends over time are similar (Fig 1).

### Smoking amount and histologic subtypes

Among ever smoking female lung cancer patients, just over half of patients’ (51.7%) had over 20 pack years smoking history. While the proportion of those with 10–19 pack years smoking history appeared to decrease over time (from 22% to 14.4%) between 2000–2004 and 2009–2012, the proportion with 20 or more pack years appeared to increase (from 48% to 56%), although these changes were not statistically significant. Histology also varied by the extent of smoking history. Among the smoking cases with ADC, the proportion with less than 10 pack years of smoking history decreased over time relative to other subtypes (from 40.9% to 36.8%), while the proportion for those with 20 pack years or more increased (from 36.4% to 50.3%). Similarly, among smoking SCLC cases, the proportion with less than 10 pack years smoking history decreased (from 26.3% to 9.1%) while the proportion with 20 or more years increased (from 57.9% to 70.9%, *P* = 0.045) (Table 2).

**Table 2:** Distribution of smoking amount by histologic subtype and time

Smoking amount/histology	N	%	2000–2004		2005–2008		2009–2012			
			N	%	N	%	N	%	*X* ^2^	*P*
All	630		123		209		298		5.85	0.211
<10	194	30.8	37	30.1	69	33.0	88	29.5		
10–19	110	17.5	27	22.0	40	19.1	43	14.4		
>20	326	51.7	59	48.0	100	47.8	167	56.0		
SCC	183		49		67		67		2.61	0.625
<10	40	21.9	8	16.3	16	23.9	16	21.9		
10–19	36	19.7	13	26.5	12	17.9	11	16.4		
>20	107	58.5	28	57.1	39	58.2	40	59.7		
ADC	295		44		96		155		6.63	0.157
<10	111	37.6	18	40.9	36	37.5	57	36.8		
10–19	52	17.6	10	22.7	22	22.9	20	12.9		
>20	132	44.7	16	36.4	38	39.6	78	50.3		
SCLC	114		19		40		55		9.75	0.045
<10	24	21.1	5	26.3	14	35.0	5	9.1		
10–19	20	17.5	3	15.8	14	15.0	5	20.0		
>20	70	61.4	11	57.9	20	50.0	39	70.9		

ADC, adenocarcinoma; SCC, squamous cell carcinoma; SCLC, small cell lung cancer; the pack‐year is the average daily packs multiplied by the total years smoked.

### Smoking status and histologic subtypes by eighthth AJCC stage

Among SCC patients, smoking patients were more likely diagnosed at early stages compared with non‐smoking patients. While almost half (47.1%) of SCC patients diagnosed at stage I/II were smokers, the proportion of smokers was much lower among those diagnosed at later stages (34.3% at Stage III; 18.6% at Stage IV). However, for ADC and SCLC patients, the proportions of smoking and non‐smoking cases were not statistically different by stage (Table 3).

**Table 3:** Smoking distribution in major histologic subtypes by staging

	I/II		III		IV		Unknown		*X* ^2^	*P*
	No.	%	No.	%	No.	%	No.	%		
ADC									*5.72*	*0.126*
Smoking										
YES	99	6.5	71	8.6	54	5.9	71	6.9		
NO	1420	93.5	750	91.3	863	94.1	953	93.1		
SCC									*15.57*	*<0.001*
Smoking										
YES	72	47.1	48	34.3	11	18.6	52	37.1		
NO	81	52.9	92	65.7	48	81.4	88	62.9		
SCLC	Limited			Extensive			Unknown		*0.36*	*0.834*
Smoking	No.	%		No.	%		No.	%		
YES	47	17.7		22	15.9		45	18.4		
NO	219	82.3		116	84.1		200	81.6		

ADC, adenocarcinoma; SCC, squamous cell carcinoma; SCLC, small cell lung cancer.

### Residential area and histology subtypes

When patients without smoking were categorized by their home province, a trend of increasing ADC and decreasing SCC from 2000–2004 to 2009–2012 was evident for each province represented in the data, though not all of these differences were statistically significant. These trends in changing histologic subtypes over time were significant for Beijing, Hebei, Shandong, and Liaoning provinces. For example, for Beijing, which accounted for the largest number of patients, the proportion of ADC increased from 70.9% in 2000–2004 to 84.0% in 2009–2012 while SCC decreased from 11.5% in 2000–2004 to 3.90% in 2009–2012 (Table 4).

**Table 4:** Distribution of major histologic subtypes in non‐smokers by residential area and time

Provinces	N		2000‐2004		2005‐2008		2009‐2012			
			N	%	N	%	N	%	*X* ^*2*^	*P*
Beijing	1828	SCC	41	11.5	30	5.1	34	3.9	*38.99*	*<0.001*
		ADC	253	70.9	482	81.4	738	84		
		SCLC	34	9.5	41	6.9	48	5.5		
		Other	29	8.1	39	6.6	59	6.7		
Hebei	754	SCC	11	10.9	12	5.3	16	3.7	*17.80*	*0.007*
		ADC	53	52.5	161	71.2	299	70		
		SCLC	26	25.7	40	17.7	76	17.8		
		Other	11	10.9	13	5.8	36	8.4		
Inner M	424	SCC	3	5.6	6	4.7	11	4.6	*10.16*	*0.118*
		ADC	31	57.4	89	69.0	179	74.3		
		SCLC	9	16.7	23	17.8	30	12.4		
		Other	11	20.4	11	8.5	21	8.7		
HLJ	354	SCC	4	8.2	8	7.1	13	6.7	*9.82*	*0.133*
		ADC	34	69.4	80	71.4	160	82.9		
		SCLC	5	10.2	13	11.6	12	6.2		
		Other	6	12.2	11	9.8	8	4.1		
Liaoning	352	SCC	6	16.2	13	13.1	13	6	*13.79*	*0.032*
		ADC	22	59.5	70	70.7	174	80.6		
		SCLC	4	10.8	11	11.1	20	9.3		
		Other	5	13.5	5	5.1	9	4.2		
Shandong	342	SCC	9	17.0	3	3.1	5	2.6	*21.20*	*0.002*
		ADC	35	66.0	73	76.0	158	81.9		
		SCLC	5	9.4	12	12.5	15	7.8		
		Other	4	7.5	8	8.3	15	7.8		
Other	1186	SCC	13	8.7	28	8.6	30	4.2	*16.05*	*0.013*
		ADC	105	70.0	238	73.0	552	77.7		
		SCLC	13	8.7	37	11.3	61	8.6		
		Other	19	12.7	23	7.1	67	9.4		

ADC, adenocarcinoma; SCC, squamous cell carcinoma; SCLC, small cell lung cancer; Other: the group of other included subtypes of large cell carcinoma (LCC), adenosquamous carcinoma (ASC) and other specified; Inner M, Inner Mongolia; HLJ, Heilongjiang.

## Discussion

The vast majority (89.3%) of lung cancers seen among female patients at the CHCAMS were in non‐smokers. This is in contrast to previous results among men, where the majority of male patients were smokers.^16^This result may appear surprising given the strong association of smoking with lung cancer but is explained by the very low female smoking prevalence in China and the presence of other risk factors. Non‐smoking cases tended to be younger compared with the smoking cases. This could be due to a difference in lung cancer type or etiology or it could be due to smoking patterns by age (older women may be more likely to smoke).

While there were relatively few female smokers in this study, it is likely that many more subjects were regularly exposed to secondhand smoke, given the high smoking prevalence among Chinese men and limited coverage of smoke free policies in China until very recently.^19^, ^20^, ^21^A recent analysis reported that secondhand smoke exposure accounted for 50% of all tobacco‐related cancer deaths (a large proportion of which were lung cancers) among women in 2014.^22^ In general, smoking is much less accepted for women in China and smoking prevalence among women remains relatively low, especially in more traditional, rural areas in the south of China.^23^ However, smoking among women is higher in Northeast China and in urban areas like Beijing and Tianjin, where female smoking is more socially acceptable.^24^

While squamous cell carcinoma is almost exclusively linked to cigarette smoking, adenocarcinoma has multiple causes (including air pollution and cigarette smoking). Additionally, the relative risks differ substantially for different lung cancer subtypes in relation to tobacco exposure.^25^ However, adenocarcinoma is also strongly linked with cigarette smoking.^26^Moreover, a similar shift in lung cancer histology was seen earlier in the U.S. and European countries.^27^Epidemiologic studies suggest that the increase in adenocarcinoma in Western countries is due to changes in cigarette design and smoking behavior. During the 1960s and 1970s, tobacco companies increasingly marketed “light” and low‐tar cigarette brands with lower machine‐measured levels of tar and nicotine, and these brands came to dominate the market in large part due to the perception that they were less harmful than other cigarettes. As smokers switched to low‐tar cigarettes, they tended to inhale more deeply, transporting carcinogens more distally into the lungs where adenocarcinomas arise. At the same time, greater use of reconstituted tobacco, with higher concentrations of nitrosamines, may have also contributed to a shift towards adenocarcinomas.^28^However, China has more recently experienced a similar shift towards ‘low tar’ cigarettes. Average machine‐measured tar content per cigarette decreased from about 27 milligrams in 1983 to 17 milligrams in 2000 and 12 milligrams in 2010.^14^ Thus, it is likely that the increase in adenocarcinomas relative to other lung cancer subtypes is attributable, at least in part, to changes in cigarette design and smoking behavior.

Understanding trends of lung cancer and tobacco smoke exposure in China is also complicated by the role of competing risks, particularly indoor and outdoor air pollution. In particular, high lung cancer mortality among non‐smoking women in China has been attributed to household air pollution from cooking and the use of coal for heating.^29^Lung cancer among women in China has historically been higher in the northeast of the country, where indoor heating exposure would be expected to be higher.^30^

This study has some limitations that are important to note. These findings represent results from a single hospital, so are not necessarily representative of the entire population. However, as the primary national cancer hospital in the country, the hospital receives patients from other regions as well as Beijing. Additionally, patient data did not include information on secondhand smoke exposure or other potential risk factors, such as air pollution. Nevertheless, given the strong effect of tobacco smoking, differences were still observed in this group.

The tobacco epidemic remains at an earlier stage in China compared with North America and Europe, and the full impact of tobacco smoking patterns in recent decades on cancer mortality may not yet have been realized.^31^ There is a substantial lag time between tobacco use and cancer diagnosis or death. Thus, tobacco control measures taken now may not substantially impact cancer rates for another decade or more. Indeed, a recent analysis projected that even if all risk reduction targets are met under the United Nations Agenda for Sustainable Development, which sets a target to reduce premature mortality from non‐communicable diseases by one‐third by 2030, this goal could be met for cardiovascular disease and chronic respiratory diseases, but not for cancer.^32^ Nevertheless, if action is not taken to reduce tobacco use, the burden for cancer and other non‐communicable diseases will surely continue to grow. Thus, the sooner additional measures are taken to control tobacco use and promote tobacco cessation, the sooner a reduction in the cancer burden can be achieved.

## Conclusions

The number of female lung cancer patients has increased in CHCAMS over time. In both smoking and non‐smoking cases, the proportion of adenocarcinoma increased. Squamous cell carcinomas were more likely to be diagnosed in early stages among smokers. Understanding trends of lung cancer and tobacco smoke exposure in China is complicated by the role of competing risks, particularly indoor and outdoor air pollution, and these require further investigation.

## Disclosure

The authors declare that there are no conflicts of interest.

## References

[1] Zheng RS , Sun KX , Zhang SW *et al* [Report of cancer epidemiology in China, 2015]. Zhonghua Zhong Liu Za Zhi 2019; 41: 19–28. 30678413 10.3760/cma.j.issn.0253-3766.2019.01.005

[2] Chen W , Zheng R , Baade PD *et al* Cancer statistics in China, 2015. CA Cancer J Clin 2016; 66: 115–32. 26808342 10.3322/caac.21338

[3] Bray F , Ferlay J , Soerjomataram I , Siegel RL , Torre LA , Jemal A . Global cancer statistics 2018: GLOBOCAN estimates of incidence and mortality worldwide for 36 cancers in 185 countries. CA Cancer J Clin 2018; 68: 394–424. 30207593 10.3322/caac.21492

[4] Zou XN . Epidemic trend, screening, and early detection and treatment of cancer in Chinese population. Cancer Biol Med 2017; 14: 50–9. 28443203 10.20892/j.issn.2095-3941.2016.0047 PMC5365185

[5] Zhi XY , Zou XN , Hu M , Jiang Y , Jia MM , Yang GH . Increased lung cancer mortality rates in the Chinese population from 1973‐1975 to 2004‐2005: An adverse health effect from exposure to smoking. Cancer 2015; 121 (Suppl 17): 3107–12. 26331817 10.1002/cncr.29603

[6] Zhang SW , Zheng RS , Yang ZX *et al* [Trend analysis on incidence and age at diagnosis for lung cancer in cancer registration areas of China, 2000‐2014]. Zhonghua Yu Fang Yi Xue Za Zhi 2018; 52: 579–85. 29886678 10.3760/cma.j.issn.0253-9624.2018.06.005

[7] Schwartz AG , Cote ML . Epidemiology of lung cancer. Adv Exp Med Biol 2016; 893: 21–41. 26667337 10.1007/978-3-319-24223-1_2

[8] Zhong L , Goldberg MS , Parent ME , Hanley JA . Exposure to environmental tobacco smoke and the risk of lung cancer: A meta‐analysis. Lung Cancer 2000; 27: 3–18. 10672779 10.1016/s0169-5002(99)00093-8

[9] Liu BQ , Peto R , Chen ZM *et al* Emerging tobacco hazards in China: 1. Retrospective proportional mortality study of one million deaths. BMJ 1998; 317: 1411–22. 9822393 10.1136/bmj.317.7170.1411 PMC28719

[10] Knoke JD , Burns DM , Thun MJ . The change in excess risk of lung cancer attributable to smoking following smoking cessation: An examination of different analytic approaches using CPS‐I data. Cancer Causes Control 2008; 19: 207–19. 17992575 10.1007/s10552-007-9086-5

[11] Wiseman M . The second World Cancer Research Fund/American Institute for Cancer Research expert report. Food, nutrition, physical activity, and the prevention of cancer: A global perspective. Proc Nutr Soc 2008; 67: 253–6. 18452640 10.1017/S002966510800712X

[12] Liu S , Zhang M , Yang L *et al* Prevalence and patterns of tobacco smoking among Chinese adult men and women: Findings of the 2010 national smoking survey. J Epidemiol Community Health 2017; 71: 154–61. 27660401 10.1136/jech-2016-207805 PMC5284482

[13] U.S. National Cancer Institute and World Health Organization. *The Economics of Tobacco and Tobacco Control*. National Cancer Institute Tobacco Control Monograph21. NIH Publication. Bethesda, MD, U.S. 2016.

[14] Zou XN , Lin D , Chao A *et al* Histological subtypes of lung cancer in Chinese women from 2000 to 2012. Thorac Cancer 2014; 5: 447–54. 26767037 10.1111/1759-7714.12121 PMC4704366

[15] Zou XN , Lin DM , Wan X *et al* Histological subtypes of lung cancer in Chinese males from 2000 to 2012. Biomed Environ Sci 2014; 27: 3–9. 24553368 10.3967/bes2014.010

[16] Jia M , Li J , Lin H , Zou X , Zhao P . [Effect of smoking on lung cancer histology and its epidemiology in Chinese male]. Zhongguo Fei Ai Za Zhi 2017; 20: 516–21. 28855031 10.3779/j.issn.1009-3419.2017.08.03 PMC5973006

[17] Travis WD , Brambilla E , Burke AP , Marx A , Nicholson AG . WHO Classification of Tumours of the Lung, Pleura, Thymus and Heart, 4th edn. IARC Publications, Lyon 2015. 10.1097/JTO.0000000000000663 26291007

[18] Amin MB , Edge S , Greene F *et al* *AJCC Cancer Staging Manual. Eighth Edition* Springer International Publishing, New York 2017.

[19] Yang G , Wang Y , Wu Y , Yang J , Wan X . The road to effective tobacco control in China. Lancet 2015; 385: 1019–28. 25784349 10.1016/S0140-6736(15)60174-X

[20] Yang GH , Ma JM , Liu N , Zhou LN . [Smoking and passive smoking in Chinese, 2002]. Zhonghua Liu Xing Bing Xue Za Zhi 2005; 26: 77–83. 15921604

[21] Zhang M , Wang LM , Li YC *et al* [Cross‐sectional survey on smoking and smoking cessation behaviors among Chinese adults in 2010]. Zhonghua Yu Fang Yi Xue Za Zhi 2012; 46: 404–8. 22883725

[22] Xia C , Zheng R , Zeng H *et al* Provincial‐level cancer burden attributable to active and second‐hand smoking in China. Tob Control 2018; 1–7. 10.1136/tobaccocontrol-2018-054583 30322976

[23] Mao A , Bristow K , Robinson J . Caught in a dilemma: Why do non‐smoking women in China support the smoking behaviors of men in their families? Health Educ Res 2013; 28: 153–64. 22843329 10.1093/her/cys078

[24] Li Z , Yao Y , Han W *et al* Smoking prevalence and associated factors as well as attitudes and perceptions towards tobacco control in Northeast China. Int J Environ Res Public Health 2015; 12: 8606–18. 26206569 10.3390/ijerph120708606 PMC4515736

[25] Pesch B , Kendzia B , Gustavsson P *et al* Cigarette smoking and lung cancer‐‐relative risk estimates for the major histological types from a pooled analysis of case‐control studies. Int J Cancer 2012; 131: 1210–9. 22052329 10.1002/ijc.27339 PMC3296911

[26] Yang P , Cerhan JR , Vierkant RA *et al* Adenocarcinoma of the lung is strongly associated with cigarette smoking: Further evidence from a prospective study of women. Am J Epidemiol 2002; 156: 1114–22. 12480656 10.1093/aje/kwf153

[27] Thun MJ , Lally CA , Flannery JT *et al* Cigarette smoking and changes in the histopathology of lung cancer. J Natl Cancer Inst 1997; 89: 1580–6. 9362155 10.1093/jnci/89.21.1580

[28] Devesa SS , Bray F , Vizcaino AP , Parkin DM . International lung cancer trends by histologic type: Male:female differences diminishing and adenocarcinoma rates rising. Int J Cancer 2005; 117: 294–9. 15900604 10.1002/ijc.21183

[29] Xue Y , Jiang Y , Jin S , Li Y . Association between cooking oil fume exposure and lung cancer among Chinese nonsmoking women: A meta‐analysis. Onco Targets Ther 2016; 9: 2987–92. 27284248 10.2147/OTT.S100949 PMC4881732

[30] Xu ZY , Blot WJ , Fraumeni JF Jr . Geographic variation of female lung cancer in China. Am J Public Health 1986; 76: 1249–50. 10.2105/ajph.76.10.1249-a PMC1646680 3752333

[31] Torre LA , Bray F , Siegel RL , Ferlay J , Lortet‐Tieulent J , Jemal A . Global cancer statistics, 2012. CA Cancer J Clin 2015; 65: 87–108. 25651787 10.3322/caac.21262

[32] Li Y , Zeng X , Liu J *et al* Can China achieve a one‐third reduction in premature mortality from non‐communicable diseases by 2030? BMC Med 2017; 15: 132. 28693510 10.1186/s12916-017-0894-5 PMC5504650

